# Dehydration-Induced Anorexia Reduces Astrocyte Density in the Rat Corpus Callosum

**DOI:** 10.1155/2015/474917

**Published:** 2015-05-18

**Authors:** Daniel Reyes-Haro, Francisco Emmanuel Labrada-Moncada, Ricardo Miledi, Ataúlfo Martínez-Torres

**Affiliations:** Departamento de Neurobiología Molecular y Celular, Instituto de Neurobiología, Universidad Nacional Autónoma de México, Campus Juriquilla, Boulevard Juriquilla 3001, 76230 Juriquilla, QRO, Mexico

## Abstract

Anorexia nervosa is an eating disorder associated with severe weight loss as a consequence of voluntary food intake avoidance. Animal models such as dehydration-induced anorexia (DIA) mimic core features of the disorder, including voluntary reduction in food intake, which compromises the supply of energy to the brain. Glial cells, the major population of nerve cells in the central nervous system, play a crucial role in supplying energy to the neurons. The corpus callosum (CC) is the largest white matter tract in mammals, and more than 99% of the cell somata correspond to glial cells in rodents. Whether glial cell density is altered in anorexia is unknown. Thus, the aim of this study was to estimate glial cell density in the three main regions of the CC (genu, body, and splenium) in a murine model of DIA. The astrocyte density was significantly reduced (~34%) for the DIA group in the body of the CC, whereas in the genu and the splenium no significant changes were observed. DIA and forced food restriction (FFR) also reduced the ratio of astrocytes to glial cells by 57.5% and 22%, respectively, in the body of CC. Thus, we conclude that DIA reduces astrocyte density only in the body of the rat CC.

## 1. Introduction

The term anorexia describes any loss of appetite and concomitant reduction of food intake that occurs even in the presence of readily accessible food sources [1–3]. Anorexia is primarily observed in young women during adolescence, and the term anorexia nervosa applies to a psychiatric disorder that induces profound weight loss, osteoporosis and amenorrhea. The lack of effective treatments and a high mortality rate justify the use of animal models to explore the neurobiological basis of this psychiatric disorder [3]. One animal model of anorexia is dehydration-induced anorexia (DIA), which mimics the characteristic weight loss and reduced food intake observed in anorexic patients [1, 2, 4]. Thus, it is an accessible experimental model with which to explore the neurobiological origins of anorexia.

Glial cells are the major population of nerve cells in the brain and responsible for supplying metabolites to neurons [5]. Glial cells are particularly abundant in white matter tracts such as the corpus callosum (CC), where they represent more than 99% of all the somata after postnatal day 5 [6]. The CC is the main white matter tract of the brain and is involved in interhemispheric communication. The CC is composed mainly of glial cells and axons [6, 7]. Dynamic changes occur during postnatal development in the CC, and callosal axons increase from 4.4 million at birth to 11.4 million at postnatal day 5 (P5); this number is maintained until adulthood in rats [8]. However, regional differences in the splenium of the rat CC include a 15% decrease of axons from P15–P60 [9]. Accordingly, similar changes are observed for glial cell density in the three main regions of CC: genu, body, and splenium [10]. Considering all this information, our aim was to test whether glial cell density is compromised in DIA.

## 2. Materials and Methods

### 2.1. Animals and Housing

The Institutional Animal Care and Use of Laboratory Animals Committee of the UNAM Instituto de Neurobiología approved all the experimental protocols. Animals were handled in accordance with the National Institutes of Health Guide for the Care and Use of Laboratory Animals. Wistar female rats (150–180 g; 45–55 days old) were housed individually under a 12 h/12 h light-dark cycle, controlled temperature, and with food and water* ad libitum*.

### 2.2. Dehydration-Induced Anorexia

The protocol for dehydration-induced anorexia was as previously described [1, 11]. Briefly, nine animals were placed in individual cages and from them three groups of three animals each were selected. The first group received water and food* ad libitum* (control). The dehydration-induced anorexia (DIA) group received a 2.5% NaCl solution as their sole drinking liquid with no food restriction. Finally, the food-restricted (FFR) group, a positive control to distinguish between starvation and dehydration effects, received tap water* ad libitum* and the same amount of food consumed by the DIA animals. Body weight and solid food intake were recorded daily at noon for five days (Supplemental Figure 1 in Supplementary Material available online at http://dx.doi.org/10.1155/2015/474917). This experimental protocol was repeated three times (9 animals each time, total animals 27, nine per experimental group).

### 2.3. Histology

Rats were deeply anaesthetized with an overdose of sodium pentobarbital (100 mg/kg) and transcardially perfused with 100 mL of saline followed by 250 mL of chilled 4% paraformaldehyde in phosphate-buffered saline (PBS) (pH 7.4). Brains were removed, postfixed overnight, and then transferred successively to a series of sucrose solutions (from 10 to 30%). Sagittal sections (30 *μ*m) that included the whole length of the CC were obtained in a freezing microtome, and three series per animal were collected and stored in cryoprotectant solution (30% ethylene glycol/20% glycerol in PBS) at –20°C [12].

### 2.4. Immunohistofluorescence

Glial fibrillary acidic protein (GFAP) immunoreactivity was performed on floating sections [12]. Briefly, brain sections were rinsed three times in PBS buffer and then treated with 3% hydrogen peroxide for 10 min, rinsed another three times in PBS, and transferred to 1.0% sodium borohydride for 6–8 min to reduce free aldehydes. The tissue was then placed in blocking solution (5% horse serum albumin/1% Triton X-100 in PBS) for 1 h. Sections were incubated with polyclonal rabbit anti-GFAP antibody (dilution 1 : 1000, Dako-Cytomation, Fort Collins, CO, USA; 4°C, for 48 h). After washing, primary antibody was detected with Alexa 594 (1 : 500, Invitrogen) coupled to goat anti-rabbit antibody. The sections were counterstained with 4′,6-diamidino-2-phenylindole (DAPI) and mounted with Vectashield H-1000 (Vector Laboratories, Burlingame, CA, USA).

**Figure 1:**
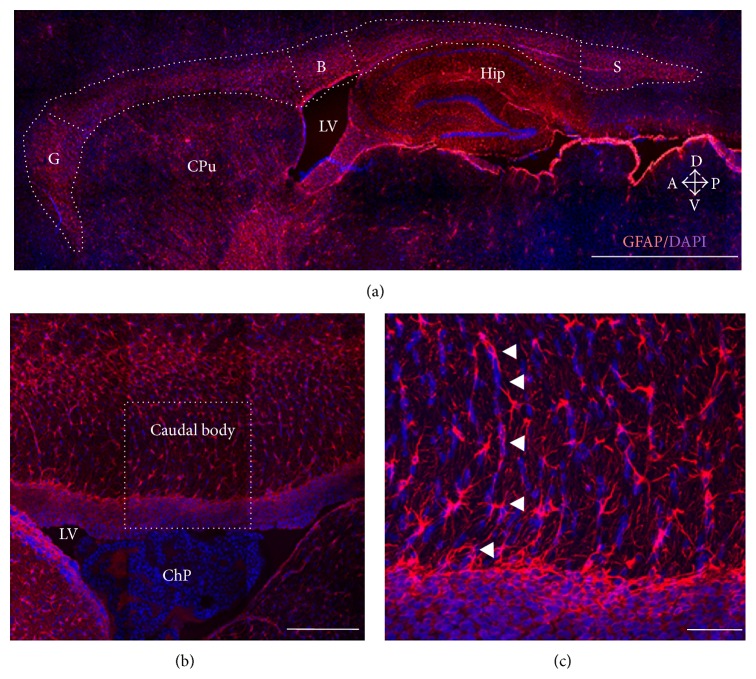
The rat corpus callosum (CC). (a) Sagittal sections from young female rat brains were immunostained for GFAP, and nuclei were labeled with DAPI. The CC was divided into three regions: genu (G), body (B), and splenium (S) to determine glial cell density. Other brain regions are indicated for anatomical reference: caudate-putamen (CPu), hippocampus (Hip), and lateral ventricle (LV). Arrows show anatomical orientation: dorsal (D), ventral (V), anterior (A), and posterior (P). (b) Higher magnification of the caudal body of the rat CC; notice that the lateral ventricle (LV) contains the choroid plexus (ChP) in close interaction with GFAP-positive cells from the CC. (c) Higher magnification of the square in (b) to highlight fibrous astrocytes from the body of the CC; notice that nuclei stained by DAPI show a radial orientation, and astrocytes align accordingly (arrowheads). Scale bars: 200 *μ*m (a), 100 *μ*m (b), and 50 *μ*m.

Brain sections containing CC were mounted on slides, photographed with a digital camera (Photometrics Cool Snap FX, USA) attached to a Nikon microscope (Nikon Eclipse E600, Tokyo, Japan), and analyzed using IMAGE J version 1.41 (NIH, Bethesda, MD, USA). A Zeiss LSM 780 Meta confocal microscope (Zeiss, Göttingen, Germany) was used for confocal images with Alexa 594 (excitation/emission wave length 590/617 nm) and DAPI (excitation/emission wave length 350/460 nm) (Figure 1).

### 2.5. Glial Cell Counting and Area Estimation

#### 2.5.1. Glial Cell Counting

The population of GFAP-positive cells was compared to the number of DAPI-labeled nuclei in each slide and each CC region as previous described [10]. Briefly, three tissue sections from nine animals per group were used for cell counting. A test square grid of 0.01 mm^2^ in each section was used to determine the number of DAPI-stained nuclei (equivalent to the total number of glial cells) and GFAP^+^ cells (corresponding to astrocytes) per mm^2^; only process-bearing cells with evident soma in the plane of the analyzed area were counted. Six to eight randomly chosen fields were counted by progressive displacement of the test square grid. The astrocyte/glial cell ratio results from the estimated number of GFAP^+^ cells divided by the total number of glial cells labeled with DAPI.

#### 2.5.2. Average Astrocyte Area

CellProfiler software was used to estimate the area of cells [13]. Briefly, astrocytes were identified as primary objects. The identification considered the size and the shape of the cells, as well as the intensity and background of the image. Astrocyte area was automatically estimated for each cell and the corresponding values were obtained in Excel for analysis. Finally, average astrocyte area was calculated for control, DIA, and FFR experimental groups.

The data are presented as the mean ± standard error of the mean (S.E.M.). Statistical analysis of data was performed by using a one-way ANOVA followed by a Bonferroni posttest with Origin 7.0 software. Differences with *P* < 0.05 were considered statistically significant.

## 3. Results

### 3.1. Dehydration-Induced Anorexia (DIA) Has No Effect on the Density of Nuclei

**Figure 2:**
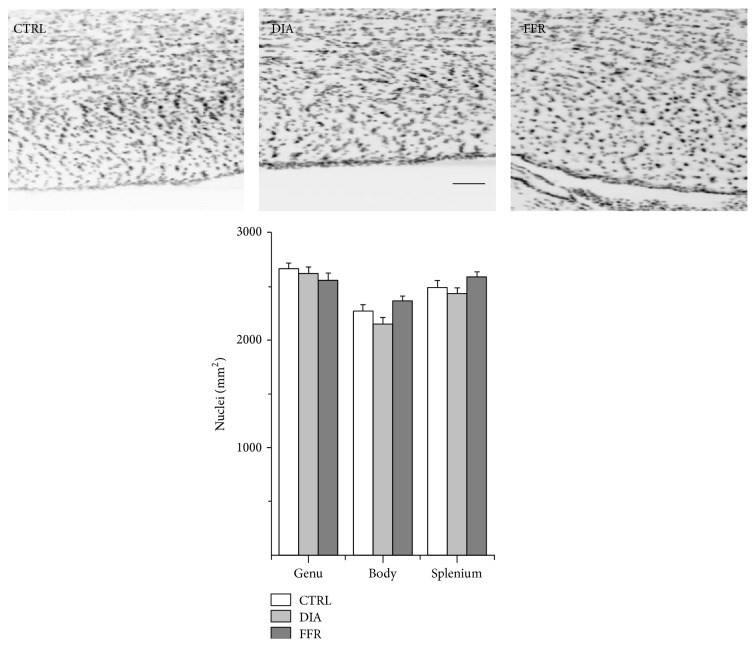
Dehydration-induced anorexia (DIA) has no effect on glial cell density. Sagittal sections of the body of the rat CC show DAPI staining in control (CTRL), dehydration-induced anorexia (DIA) and forced food-restricted (FFR) groups. The density of nuclei did not differ significantly among the three experimental groups in the genu (*n* ≥ 23), body (*n* ≥ 26), or splenium (*n* ≥ 20). Scale bar = 100 *μ*m. Data are mean ± S.E.M.

Glial cell density in the genu was 2655 ± 64 nuclei/mm^2^ (*n* = 26) in the control group; this did not change significantly in either the DIA group (2607 ± 75 nuclei/mm^2^ (*n* = 23)) or the FFR group (2547 ± 81 nuclei/mm^2^ (*n* = 24)) (*P* = 0.574) (Figure 2, Table 1). Similar results were observed for the body of the CC, where the estimated glial cell density for the control group was 2261 ± 69 nuclei/mm^2^ (*n* = 26), while for DIA it was 2140 ± 71 nuclei/mm^2^ (*n* = 28) and for FFR it was 2359 ± 53 nuclei/mm^2^ (*n* = 26) (*P* = 0.061) (Figure 2, Table 1). The splenium showed no significant changes among the control (2480 ± 76 nuclei/mm^2^ (*n* = 20)), DIA (2418 ± 70 nuclei/mm^2^ (*n* = 26)), and FFR (2580 ± 57 nuclei/mm^2^ (*n* = 26)) (*P* = 0.207) (Figure 2, Table 1). Thus, we conclude that the cell density of the CC region is not altered in either the DIA or FFR.

### 3.2. Dehydration-Induced Anorexia (DIA) Reduces Astrocyte Density in the Body of Rat CC

**Figure 3:**
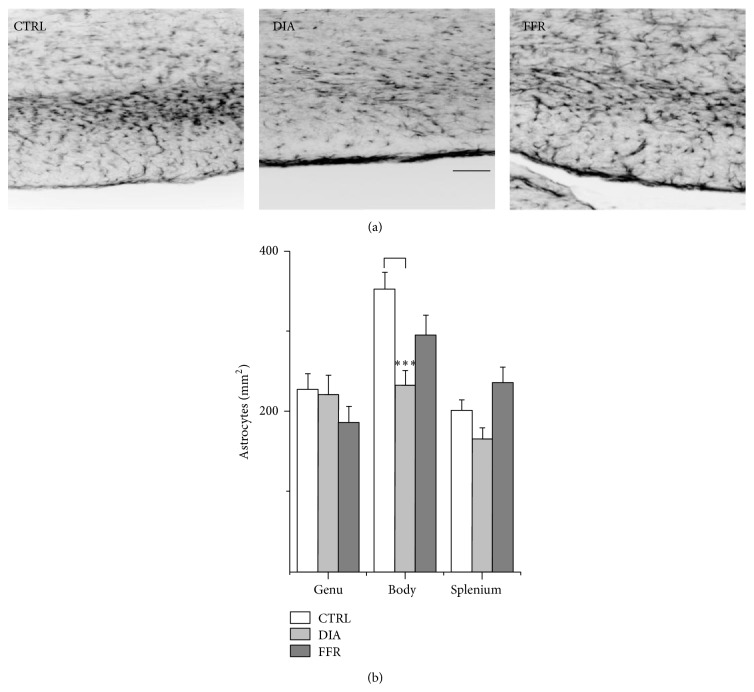
Dehydration-induced anorexia (DIA) reduces astrocyte density in the body of rat CC. (a) Histological sections of the body of rat CC show GFAP staining in control (CTRL), dehydration-induced anorexia (DIA), and forced food-restricted (FFR) groups. (b) No differences in astrocyte density between control, DIA, or FFR groups were found in the genu (*n* ≥ 23) or the splenium (*n* ≥ 20). In contrast, a significant reduction in astrocyte density was observed in the caudal body (*n* ≥ 26) for the DIA group (−34%). Scale bar = 100 *μ*m. Data are mean ± S.E.M. (^∗∗∗^ *P* < 0.001).

In sharp contrast to the unaffected glial cell density in the DIA and FFR groups, the density of astrocytes, identified by GFAP label, was modified in both groups, and the effect was different among the three regions of the CC. In the genu of the control group, the density of GFAP^+^ cells was 227 ± 20 astrocytes/mm^2^ (*n* = 26), while for the DIA and FFR groups no significant changes were observed [220 ± 25 (*n* = 23) and 186 ± 20 (*n* = 24), respectively (*P* = 0.362)] (Figure 3, Table 1). On the other hand, the caudal body of the CC that faces the lateral ventricles showed a higher density of astrocytes in the control group (352 ± 22 astrocytes/mm^2^; *n* = 26) than in the DIA group, where a reduction of 34% was estimated (232 ± 19 astrocytes/mm^2^; *n* = 27; *P* = 0.00095); no significant changes were observed for the FFR group (295 ± 25 astrocytes/mm^2^; *n* = 26; *P* = 0.091) (Figure 3, Table 1). Finally, we estimated the astrocyte density for the splenium, and no significant differences were found among the control, DIA, and FFR groups (200 ± 14 (*n* = 20), 164 ± 15 (*n* = 20), and 234 ± 21 (*n* = 26) astrocytes/mm^2^; *P* = 0.205). Interestingly, a significant difference was detected in astrocyte density between the DIA and FFR groups (*P* = 0.016) (Figure 3, Table 1); therefore, we conclude that in the DIA group, a reduction in astrocyte density occurs in the body of the CC.

### 3.3. Astrocyte/Glial Cell Ratio Is Reduced in the CC Body of DIA and FFR Groups

**Figure 4:**
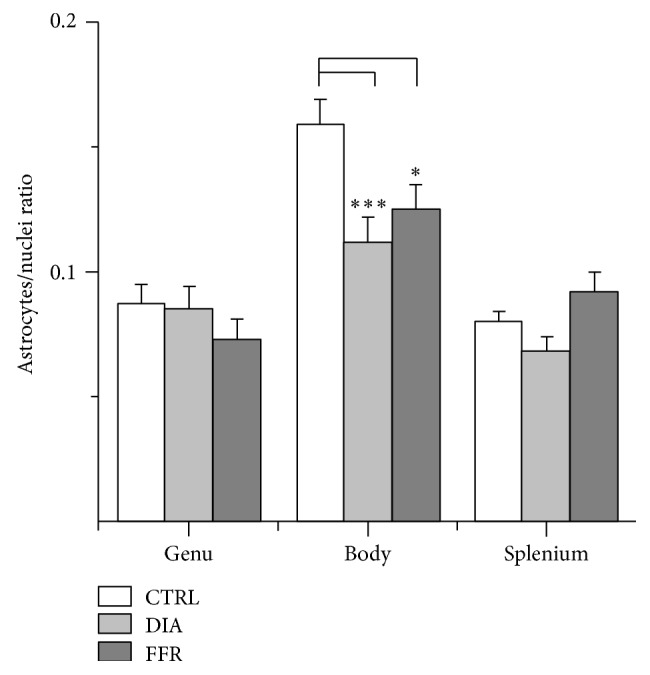
Astrocyte/glial cell ratio is reduced in the body of the DIA and FFR groups. The astrocyte/nuclei ratio was significantly reduced in both the dehydration-induced anorexia (DIA) and forced food-restricted (FFR) groups only in the body of the CC (−57.5% and −22%, resp.) (*n* ≥ 26). The genu (*n* ≥ 23) and splenium (*n* ≥ 26) were not significantly affected. Data are mean ± S.E.M. (^∗∗∗^ *P* < 0.001; ^∗^ *P* < 0.05).

Our next step was to estimate the astrocyte/glial cell ratio to correlate the GFAP immunoreactivity and nuclear staining. This is a more accurate estimate since it considers the glial cell density which is known to vary among regions [10]. The astrocyte/glia ratio for the genu in the control group was 0.087 ± 0.008 (*n* = 26), and no significant differences were observed for DIA (0.085 ± 0.009; *n* = 23) and FFR (0.073 ± 0.008; *n* = 24) (*P* = 0.476) (Figure 4). In the body of CC, the astrocyte/glia ratio was 0.160 ± 0.010 (*n* = 26), but it was significantly reduced in the DIA (−57.5%; 0.068 ± 0.005; *n* = 27) and FFR groups (−22%; 0.125 ± 0.010; *n* = 26) (*P* = 0.023) (Figure 4). Finally, the astrocyte/glia ratio for the splenium was 0.080 ± 0.004 (*n* = 20) for the control group, while for the DIA (0.068 ± 0.005; *n* = 25) and FFR (0.092 ± 0.008; *n* = 26) (*P* = 0.26). A significant change was also observed between the DIA and FFR groups (*P* = 0.047) (Figure 4). Thus, we conclude that astrocyte/glia ratio is reduced by DIA and FFR in the body of the rat CC.

The Average Astrocyte Area Remains Constant in DIA and FFR Groups

**Figure 5:**
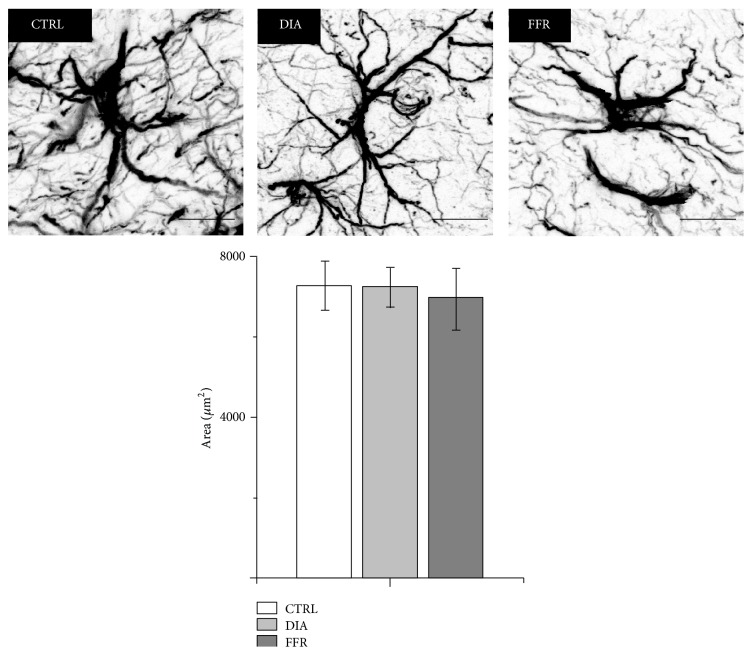
The average astrocyte area remains constant in DIA and FFR groups. The average area of astrocytes was estimated for control (*n* = 799), DIA (*n* = 1201), and FFR (*n* = 806) groups. No significant changes were observed. Scale bar = 10 *μ*m. Data are mean ± S.E.M.

Neuropathological disorders such as anorexia could modify regular morphology of astrocytes. We estimated if morphology of astrocytes is affected in terms of area. The average area of astrocytes in control animals was 7, 287 ± 184 *μ*m^2^ (*n* = 799 astrocytes, from 11 histological sections). This value was not significantly different from DIA (7, 237 ± 127 *μ*m^2^; *n* = 1201 astrocytes from 15 histological sections) or FFR (6, 941 ± 245 *μ*m^2^; *n* = 806 astrocytes from 10 histological sections) (*P* = 0.388) (Figure 5). Thus, we conclude that there are no significant changes in the average area of astrocytes from control, DIA, or FFR animals. Nevertheless, morphological changes or overlap in astrocyte domains were occasionally observed in some histological sections of DIA animals (data not shown).

## 4. Discussion

Dehydration, malnutrition, and anorexia alter the homeostasis of the central nervous system. Indeed, brain dehydration induces a reduction in blood volume (hypovolaemia), an increase in serum osmolality (hypernatremia) and cerebrospinal fluid volume in the ventricles, and a decrease in tissue volume of white matter in the CC [14, 15]. The CC is the main white matter tract in mammals, and a possible correlation between decreased volume and histological modifications in glial density might occur during dehydration-induced anorexia. In support of this hypothesis, prenatal malnutrition affects the diameter of both myelinated and unmyelinated fibers. Moreover, an increased density of unmyelinated fibers is observed in the splenium of the CC [16]. Interestingly, the model of DIA provokes a voluntary reduction of food intake that results in acute weight loss due to dehydration [1, 2, 4, 11]. Thus, we tested if DIA affected glial cell density in the rat CC. We used a group of FFR rats to distinguish between dehydration and anorexia effects on glial cell density (see Section 2). Our results showed that DIA reduced astrocyte density of the body (−57.5%) in the middle region of CC, while no significant changes were observed in the genu or the splenium. Our results are in agreement with a previous study where a reduction in cell proliferation was reported for the medial CC in activity-based anorexia (ABA), another model that mimics the characteristics of anorexia nervosa [3]; however, the cell identity of the reduced population was not investigated in this ABA model. Thus, we conclude that astrocyte density is significantly reduced in the body of the CC in both DIA and FFR. This reduction was specifically observed in the caudal region that faces the lateral ventricles.

Astrocytes are key elements for metabolism, blood flow, and water homeostasis of the brain [17]. There is no clear association of the CC with psychiatric behavior related to anorexia. The DIA model is a first approximation to investigate if any cellular changes occur in this region. Interestingly, we found that astrocyte density is reduced in the CC of DIA animals, particularly in the caudal region of the body. This result could reflect an imbalance in astrocyte population dynamics. In support of this hypothesis, a recent study of genetic fate-mapping revealed that a continuous turnover of astrocytes occurs in adult CC. The entry of new astrocytes, generated from neuronal progenitor cells from the subventricular zone (NPCs-SVZ), is balanced by astroglial apoptosis [18]. We propose that DIA model could modify this balance with a turnover insufficient to overcome astrocyte apoptosis rate. Another possibility is that apoptosis is upregulated in DIA model producing a deficit in astrocyte turnover rate. Further studies in other brain regions will show whether astrocyte density is also reduced in anorexia. An important conclusion is that our results with the DIA model are similar to previous observations with the ABA model. This information will provide a better understanding of the effects of anorexia behavior on brain function.

## Supplementary Material

Body weight and food intake in DIA and FFR animals.
The food intake and body weight of the rats was monitorated daily for each experimental group, during five days. DIA animals decreased their food intake since the first day and this was consistently reflected in the body weight of the animals. The FFR group received the same amount of food ingested by DIA animals, thus the curves describing food intake and weight were similar. Notice that the food intake remains constant for the control group, while the body weight shows a daily increase.

## Figures and Tables

**Table 1:** Regional changes in glial cell density by DIA and FFR.

	Control	DIA	FFR	*P*
Glial cells				
Genu	2655 ± 64 (*n* = 26)	2607 ± 75 (*n* = 23)	2547 ± 81 (*n* = 24)	0.574
Body	2261 ± 69 (*n* = 26)	2140 ± 71 (*n* = 28)	2359 ± 53 (*n* = 26)	0.061
Splenium	2480 ± 76 (*n* = 20)	2418 ± 70 (*n* = 26)	2580 ± 57 (*n* = 26)	0.207

Astrocytes				
Genu	227 ± 20 (*n* = 26)	220 ± 25 (*n* = 23)	186 ± 20 (*n* = 24)	0.362
Body	352 ± 22 (*n* = 26)	232 ± 19^∗∗∗^ (*n* = 27)	295 ± 25 (*n* = 26)	<0.001
Splenium	200 ± 14 (*n* = 20)	164 ± 15 (*n* = 25)	234 ± 21 (*n* = 26)	0.091

Astrocytes/glia ratio				
Genu	0.087 ± 0.008 (*n* = 26)	0.085 ± 0.009 (*n* = 23)	0.073 ± 0.008 (*n* = 24)	0.476
Body	0.160 ± 0.010 (*n* = 26)	0.112 ± 0.010^∗∗∗^ (*n* = 27)	0.125 ± 0.010^∗^ (*n* = 26)	<0.05
Splenium	0.080 ± 0.004 (*n* = 20)	0.068 ± 0.005 (*n* = 25)	0.092 ± 0.008 (*n* = 26)	0.26

Data are mean ± S.E.M. (^∗∗∗^ *P* < 0.001; ^∗^ *P* < 0.05).

## References

[1] Watts A. G. (1998). Dehydration-associated anorexia: development and rapid reversal. *Physiology & Behavior*.

[2] Watts A. G., Boyle C. N. (2010). The functional architecture of dehydration-anorexia. *Physiology & Behavior*.

[3] Barbarich-Marsteller N. C., Fornal C. A., Takase L. F. (2013). Activity-based anorexia is associated with reduced hippocampal cell proliferation in adolescent female rats. *Behavioural Brain Research*.

[4] Jaimes-Hoy L., Joseph-Bravo P., de Gortari P. (2008). Differential response of TRHergic neurons of the hypothalamic paraventricular nucleus (PVN) in female animals submitted to food-restriction or dehydration-induced anorexia and cold exposure. *Hormones and Behavior*.

[5] Swanson R. A. (1992). Physiologic coupling of glial glycogen metabolism to neuronal activity in brain. *Canadian Journal of Physiology and Pharmacology*.

[6] Sturrock R. R. (1976). Light microscopic identification of immature glial cells in semithin sections of the developing mouse corpus callosum. *Journal of Anatomy*.

[7] Aboitiz F., Montiel J. (2003). One hundred million years of interhemispheric communication: the history of the corpus callosum. *Brazilian Journal of Medical and Biological Research*.

[8] Gravel C., Sasseville R., Hawkes R. (1990). Maturation of the corpus callosum of the rat: II. Influence of thyroid hormones on the number and maturation of axons. *Journal of Comparative Neurology*.

[9] Kim J. H. Y., Juraska J. M. (1997). Sex difference in the development of axon number in the splenium of the rat corpus callosum from postnatal day 15 through 60. *Developmental Brain Research*.

[10] Reyes-Haro D., Mora-Loyola E., Soria-Ortiz B., García-Colunga J. (2013). Regional density of glial cells in the rat corpus callosum. *Biological Research*.

[11] de Gortari P., Mancera K., Cote-Vélez A. (2009). Involvement of CRH-R2 receptor in eating behavior and in the response of the HPT axis in rats subjected to dehydration-induced anorexia. *Psychoneuroendocrinology*.

[12] Cabrera V., Ramos E., González-Arenas A., Cerbón M., Camacho-Arroyo I., Morales T. (2013). Lactation reduces glial activation induced by excitotoxicity in the rat hippocampus. *Journal of Neuroendocrinology*.

[13] Lamprecht M. R., Sabatini D. M., Carpenter A. E. (2007). CellProfiler: free, versatile software for automated biological image analysis. *BioTechniques*.

[14] Berk L., Rana S. (2006). Hypovolemia and dehydration in the oncolgy patient. *The Journal of Supportive Oncology*.

[15] Streitbürger D.-P., Möller H. E., Tittgemeyer M., Hund-Georgiadis M., Schroeter M. L., Mueller K. (2012). Investigating structural brain changes of dehydration using voxel-based morphometry. *PLoS ONE*.

[16] Olivares R., Morgan C., Pérez H. (2012). Anatomy of corpus callosum in prenatally malnourished rats. *Biological Research*.

[17] Simard M., Nedergaard M. (2004). The neurobiology of glia in the context of water and ion homeostasis. *Neuroscience*.

[18] Sohn J., Orosco L., Guo F. (2015). The subventricular zone continues to generate corpus callosum and rostral migratory stream astroglia in normal adult mice. *The Journal of Neuroscience*.

