# Supplementary figures and images for: Dehydration-Induced Anorexia Reduces Astrocyte Density in the Rat Corpus Callosum
Source: Neural Plast. 2015 May 18;2015:474917. doi: 10.1155/2015/474917 (PMC4452107; doi:10.1155/2015/474917)

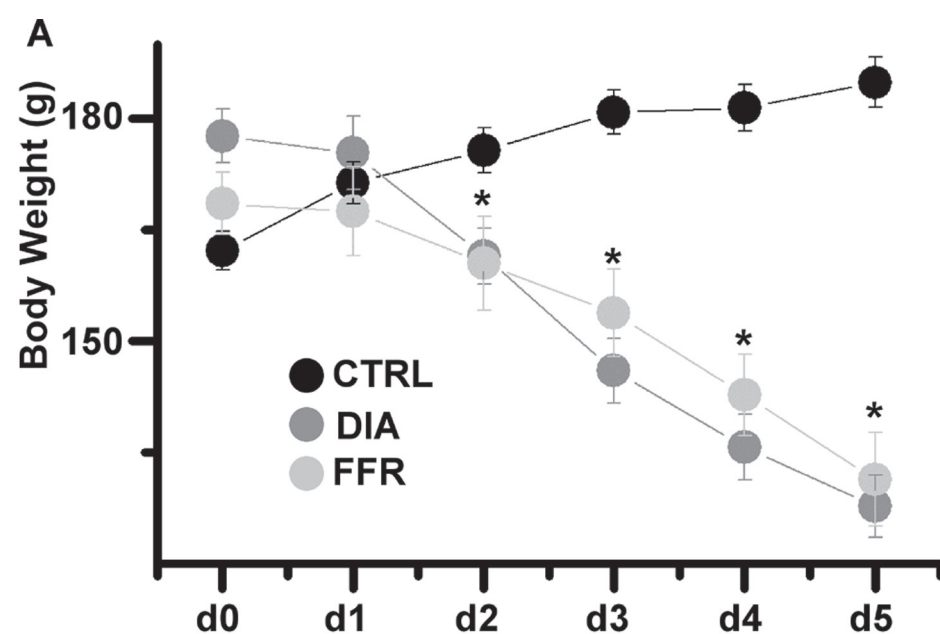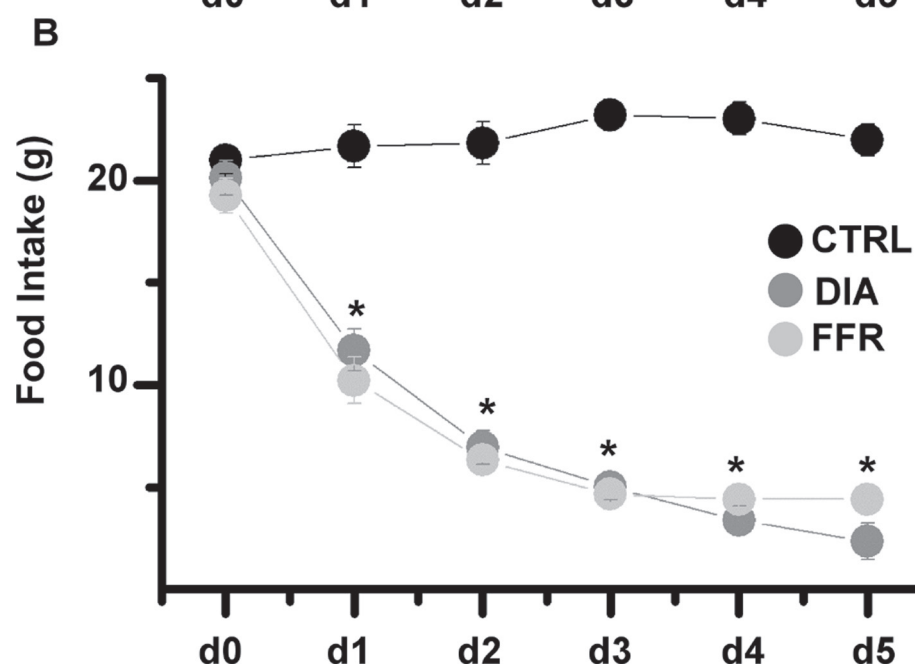

Supplement: Supplementary file 1 — Body weight and food intake in DIA and FFR animals. The food intake and body weight of the rats was monitorated daily for each experimental group, during five days. DIA animals decreased their food intake since the first day and this was consistently reflected in the body weight of the animals. The FFR group received the same amount of food ingested by DIA animals, thus the curves describing food intake and weight were similar. Notice that the food intake remains constant for the control group, while the body weight shows a daily increase. [file 474917.f1.pdf]
